# Player chronotype does not affect shooting accuracy at different times of the day in a professional, male basketball team: a pilot study

**DOI:** 10.5935/1984-0063.20220014

**Published:** 2022

**Authors:** Michael John Stacey Pengelly, Joshua H. Guy, Nathan Elsworthy, Aaron T. Scanlan, Michele Lastella

**Affiliations:** 1 Central Queensland University, School of Health, Medical and Applied Sciences - Cairns - Queensland - Australia.; 2 Central Queensland University, School of Health, Medical and Applied Sciences - Mackay - Queensland - Australia.; 3 Central Queensland University, Human Exercise and Training Laboratory - Rockhampton - Queensland - Australia.; 4 Central Queensland University, Appleton Institute for Behavioural Science - Adelaide - South Australia - Australia.

**Keywords:** Sleep, Circadian Rhythm, Team Sports, Motor Skills

## Abstract

Athlete chronotype has been documented to underpin diurnal variations in skill execution across various team sports. However, no research has explored the effects of athlete chronotype on basketball-specific skills at different times of the day. Therefore, the aim of this study was to explore diurnal variations in basketball shooting accuracy according to chronotype. Professional, male basketball players (n = 13) completed a Morningness- Eveningness Questionnaire and were categorised into chronotypes using a tertile split technique (morning-types: n = 4; neither-types: n = 4; evening-types: n = 5). Players completed separate trials of a shooting accuracy test in the morning (08:00-09:30h) and afternoon (15:00-16:30h) with each trial consisting of 20 shots attempted from four court locations at either two- or three-point distances and one-shot location from the free-throw line (100 shots in total). Each shot attempt was scored using a 0-3-point scale with higher scores awarded to more accurate shots. Non-significant (*p* >0.05) differences in shooting scores were evident between morning and afternoon trials for each chronotype group, with *small-large* effects in shooting scores favouring the morning across groups. Moreover, non-significant (*p* >0.05) differences in shooting scores were apparent between chronotype groups in the morning (*small-large* effects) and afternoon (*moderate-large* effects). Shooting accuracy appears to remain consistent across morning and afternoon performances irrespective of player chronotype in a professional basketball team, suggesting coaches may not need to schedule training sessions involving shooting tasks at specific times of the day to optimise shooting accuracy in players.

## INTRODUCTION

Chronotype is a behavioural phenotype expressing the circadian rhythmicity of an individual to indicate their inclination towards a preference for ‘morningness’ or ‘eveningness’^1,2,3^. Sleeping behaviour and diurnal activities (e.g. work, social activities) underpin the circadian rhythm of an individual resulting in a delayed or advanced acrophase (peak) in psychobiological variables (e.g. body temperature) across the day^4,5^. Variations in circadian rhythms and subsequent psychobiological acrophases across individuals led to the identification of three broad categories of chronotypes: morning types (M-types), evening types (E-types), and neither types (N-types; ^6^). The natural light/ dark cycle suits M-types who display a preference for earlier bed and wake times compared to E-types who tend to display a preference for later bed and wake times with the preferences of N-types falling between M-types and E-types^7,8^.

The circadian rhythm in psychobiological variables specific to each chronotype has been proposed to underpin diurnal variations in sports performance across athletes^9,10^. Indeed, sports performance has been shown to vary between chronotypes according to the time of day, with most research measuring physical performance (e.g. race time) in aerobic sports involving gross motor skills^2,11,12,13^. For instance, M-type rowers^12^, swimmers^2,11^, and runners^13^ have been shown to perform significantly (*p* <0.05) better in the morning compared to the evening. In contrast, N-type and E-type athletes have demonstrated diurnal variation in swimming performance with significantly (*p* <0.05) faster race times in the evening^2,11^ compared to morning. However, no differences in performance times were observed in N-type and E-type rowers^12^ between morning and afternoon performances. The varied findings observed among N-type and E-type endurance athletes may have been due to the time-of-day at which performance was assessed in previous studies. Specifically, N-type and E-type swimmers were assessed at 18:30-19:00h compared to morning (06:30-07:00h; ^2,11^). Conversely, N-type and E-type rowers^12^, demonstrated consistent performances when assessed between 05:00-07:00h and 16:30- 18:00h. Consequently, greater discrepancies in aerobic performance involving gross motor skills may occur when morning assessments are compared to assessments conducted later in the day, which coincide with proposed peaks in body temperature (~17:30-20:00h;^9,14^).

Unlike gross motor skills, performance of fine motor skills reliant on cognitive and sensory-motor components has been shown to peak in both the afternoon and evening compared to the morning across a range of sporting tasks^9,10,15,16,17^. For example, tennis first serve velocity ([16:30- 18:00h]; ^15,16^), badminton serve accuracy ([14:00h]; ^17^), and soccer volleying, chipping ([16:00h, 19:00-21:00h];^9,10^), and dribbling^9^ performance have been shown to be superior later in the day compared to morning (i.e. 07:00-09:00h). However, the applicability of current data regarding diurnal variations in sport-specific skills according to chronotype are limited due to research examining skill-based performances exclusively in one chronotype group rather than across multiple chronotype groups^9,10,15,17^. Further research is therefore warranted to determine the effect of athlete chronotype on sport-specific skill performance across different times of the day.

While data are available demonstrating the effect of diurnal variation on the performance of sports- specific skills across different times of the day^9,10,15,16,17^, no studies have specifically examined basketball skills. Basketball is a global team sport that involves execution of various skills during game-play^18^. Among the varied skills performed in basketball, shooting accuracy is a strong determinant (two-point field goal percentage: R^2^ = 0.45; three-point field goal percentage: R^2^ = 0.60) of team ranking in professional basketball players^19^. In this regard, examining diurnal variations in basketball shooting accuracy according to chronotype is important given players typically train and compete at different times of the day^20,21^. Identifying diurnal variations in shooting accuracy according to chronotype can inform coaching staff on the prescription of training schedules to individually optimise shooting performance across players. Therefore, the aims of this study were to: 1) examine diurnal variations in shooting accuracy according to player chronotype and 2) compare shooting accuracy between chronotypes at different times of the day.

## MATERIAL AND METHODS

### Participants

Basketball players (n = 13) were recruited from the same basketball team registered in the National Basketball League (NBL), which is highest professional basketball competition in Australia. All playing positions were represented among the sample including guards (n = 7), forwards (n = 4), and centres (n = 2). Descriptive statistics of the players according to chronotype group are presented in Table 1. All players showed no signs or symptoms of any sleep disorder prior to participating using the Pittsburgh Sleep Quality Index (global sleep qualtiy index: 1-7; 22). All players anywhere free from injury or illness and provided informed written informed consent prior to participating. This study was approved by an institutional ethics committee (approval no: 21175).

**Table 1:** Median (inter-quartile range) player characteristics according to chronotype group.

Characteristic	Chronotype Group			All players (n = 13)
	M-type (n = 4)	N-type (n = 4)	E-type (n = 5)	
Age (yr)	29.0 (25.8 – 32.5)	24.5 (24.0 – 25.5)	22.0 (20.0 – 23.0)	25.0 (23.0 – 27.0)
Height (cm)	197.0 (187.5 – 207.0)	197.5 (193.0 – 202.8)	192.0 (187.0 – 195.0)	193.0 (188.0 – 204.0)
Body mass (kg)	85.5 (84.5 – 97.0)	103.5 (97.0 – 111.8)	81.0 (80.0 – 90.0)	90.0 (83.0 – 100.0)
Professional playing experience (yr)	8.0 (5.5 – 11.3)	4.0 (3.3 – 4.8)	2.0 (1.0 – 5.0)	4.0 (2.0 – 7.0)
Morningness- eveningness questionnaire score	62.0 (58.0 – 67.5)	51.5 (50.3 – 52.3)	45.0 (42.0 – 45.0)	51.0 (45.0 – 55.0)
Guards (n)	2	2	3	7
Forwards (n)	1	1	2	4
Centres (n)	1	1	0	2

### Shooting accuracy tests

**Figure 1:**
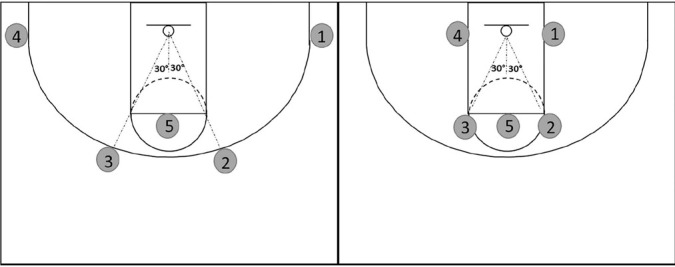
Shooting locations for perimeter (left) and non-perimeter (right) shooting players during the modified Basketball Jump Shooting Accuracy Test.

Two shooting protocols were employed separately for perimeter and non-perimeter shooting players (Figure 1). Perimeter shooting players (n = 11) were identified by the team head coach as adept at attempting three-point field goals while non-perimeter shooting players (n = 2) were identified as inept at attempting three-point field goals. The two shooting protocols were modified versions of the Basketball Jump Shooting Accuracy Test (BJSAT). The BJSAT has demonstrated suitable content validity (two-point vs. three-point shooting scores: *p* <0.01) and retest reliability (two-point shooting score: intraclass correlation coefficient [ICC] = 0.68; three-point shooting score: ICC = 0.58) in assessing basketball shooting accuracy in semi-professional, male and female basketball players^23,24^. Non-perimeter shooting players completed shots from five locations, including four locations around the key and one location at the centre of the free-throw line (Figure 1). Perimeter shooting players completed shots from five locations, including four locations around the three-point arc and one location at the centre of the free-throw line (Figure 1). Players attempted 20 consecutive shots at each location before moving onto the next location in a sequential manner.

### Testing procedures

**Figure 2:**
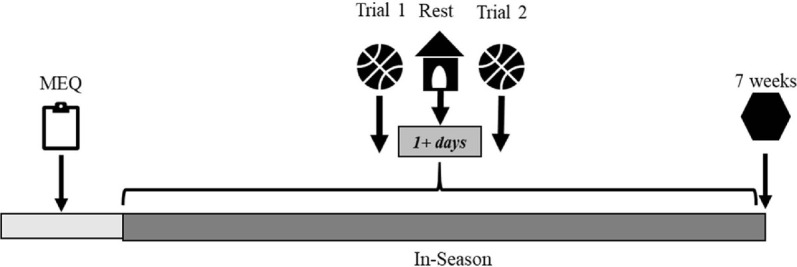
Testing procedure timeline overview.

**Figure 3:**
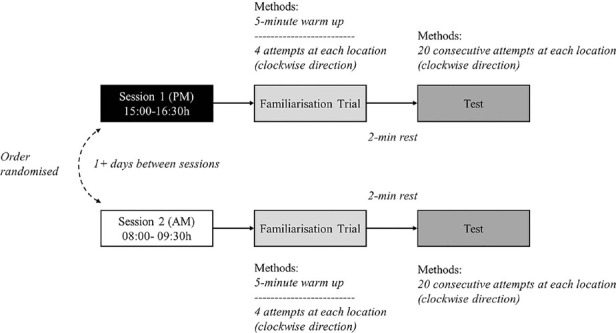
Trial procedure for morning and afternoon shooting trials.

Prior to commencing the shooting trials, players were classified into either perimeter or non- perimeter shooting players and each player completed the Morningness-Eveningness Questionnaire (MEQ) between 12:00-13:00h to determine their self-reported chronotype^1^. Each player completed two identical shooting trials on separate occasions (morning and afternoon) on the same indoor, hardwood basketball court in a randomised manner (Figure 3). Testing was conducted during the first two months of the regular season with morning sessions conducted between 08:00-09:30h and afternoon sessions between 15:00- 16:30h (Figure 2). During testing, players were undertaking 1-4 training sessions per week with each session lasting approximately 2.5 h and consisting of technical and games-based drills. In addition, the team competed in 1-2 games per week during testing. Players completed all shooting trials on any day of the week except on days prior to or following games. Morning trials were completed prior to any scheduled training sessions. Afternoon trials were completed at least 4 h following any scheduled training sessions when testing was conducted on the same day (n =7) or on days without any scheduled training (n = 6). Players completed their second testing session on a separate day as soon as possible following the completion of the first testing session (median = 1 day, interquartile range (IQR) = 1-8 days) with the number of days administered between trials dependent on game schedule and player availability^11^.

During the test protocol, shooting positions were marked on the court as 60-cm x 60-cm squares with tape. A video camera (Sony HDR- PJ410, Sony Corporation, Tokyo, Japan) was positioned on the halfway line, focused on the backboard to score shot accuracy as described in Table 2. Four different scores were able to be awarded for each shot^23^. Scores ranged from 0-3, with attempts scored retrospectively by the same assessor using captured video. Overall test performance was measured as the total summed score from all shots taken across all five positions. This scoring system has previously demonstrated strong intrarater reliability when applied in the original version of the BJSAT (Cohen’s kappa= 0.85, p <0.01; ^24^).

**Table 2:** Scoring criteria for determining shooting accuracy during the modified Basketball Jump Shooting Accuracy Test trials^23^.

Score	Description
3	Basketball travels through the basket without touching the rim or backboard
2	Basketball makes contact with the rim or backboard before travelling through the basket
1	Basketball makes contact with the rim or backboard but does not travel through the basket
0	Basketball does not make contact with the rim or backboard and does not travel through the basket

**Figure 4:**
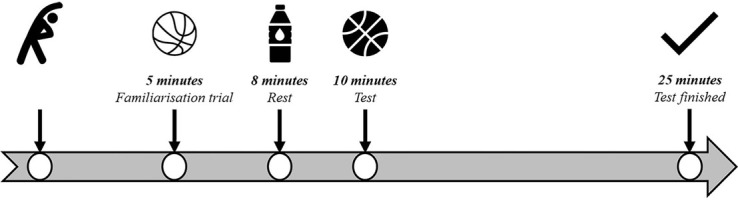
Timeline of shooting trials.

Players undertook a standardised 5-min warm-up consisting of jogging, countermovement jumps, and dynamic stretching before commencing the familiarisation trial (Figure 4). This warmup duration was elected to minimise any attenuation in diurnal variations in performance which has been observed previously with use of a 15-min warm-up^25^. Players then shot four attempts from each position in a sequential manner before undergoing 2 min of passive rest prior to trial commencement (Figure 4). Using a single standard size seven basketball to align with competition regulations (Wilson Solution; Wilson; NSW, Australia), players commenced the test from position one (Figure 1) and attempted 20 shots before moving in a clockwise direction until all positions were completed. Players received a consistent chest pass and were permitted to adjust their body position prior to attempting each shot. Players finished the test at position five by shooting 20 free- throws, resulting in a total of 100 shot attempts for the entire test. Consistent verbal instructions were provided to ensure the correct order of shooting positions was completed by each player. Across trials, the same two researchers rebounded shot attempts and passed the ball back to the player completing the test, counted shot attempts, and guided players through the test. All shots were attempted with both feet positioned within the marked square for each specific shot location. If a player attempted a shot with one foot outside of the square, verbal instruction was provided instantly to encourage the player to keep both feet within the square. Each player was encouraged to complete shot attempts at positions one to four as quickly as possible to emulate the intensity of game-play given players often have limited time to release a jump shot due to defensive pressure^26^. At position five, players were instructed to go through their typical free-throw shooting routines adopted during game-play. No time limit was placed on each trial; however, perimeter shooting players took 11.0 ± 1.2 min and non-perimeter shooting players took 9.5 ± 1.4 min to complete trials. Furthermore, time to complete morning and afternoon trials for each chronotype group remained similar (M-types, morning: 10.0 ± 1.4 min, afternoon: 10.9 ± 0.8 min; N-types, morning: 10.2 ± 1.2 min, afternoon: 10.5 ± 1.8 min; E-types, morning: 11.2 ± 0.7 min, afternoon: 11.7 ± 0.9 min).

### Morningness-Eveningness questionnaire

The MEQ is a 19-item questionnaire used to establish when the respondent feels most inclined to complete certain behaviours during a typical day^27^. Responses are assigned a value with the sum of scores ranging between 16-86^27^. The sum of scores is used to determine chronotype where M-types reflect scores ranging between 59-86, N-types between 42-58, and E-types between 16- 41^27^. In this study, three players were categorised as M-types, nine players as N-types, and one player as an E-type. To equilibrate sample sizes, the data were split into tertiles for analysis^11^, where five players were assigned to the first tertile representing the M-types (MEQ >54), four players were assigned to the second tertile representing the N-types (MEQ 47-53), and four players were assigned to the third tertile representing the E-types (MEQ <46).

### Statistical Analysis

Data were analysed using SPSS statistics (Version 25, IBM Corporation; Armonk, NY, USA). The Shapiro-Wilk test demonstrated the data were not normally distributed (*p* <0.05). Therefore, differences in shooting accuracy between morning and afternoon trials for each chronotype (M- type, N-type, and E-type) were assessed with separate Wilcoxon signed-rank tests. Differences in shooting accuracy between chronotypes were assessed with Kruskal-Wallis tests separately for the morning and afternoon trials with post hoc analyses conducted using Mann-Whitney U tests. Statistical significance for all analyses was set at *p* <0.05; however, a Bonferonni-adjusted *a*-level (*p* <0.017) was used in post hoc tests. Cohen’s R effect sizes^28^ were calculated to quantify the magnitude of pairwise differences in shooting accuracy between morning and afternoon trials for each chronotype group as well as between chronotypes in both trials, with effects interpreted as *small* (<0.30), *moderate* (0.30-0.49), or *large* (>0.50; ^29^). Given data were not normally distributed, descriptive results are expressed as the median and IQR.

## RESULTS

The median and IQR shooting scores in the morning and afternoon trials according to chronotype group are shown in Table 3. There were non-significant (*p* >0.05) differences in shooting scores between morning and afternoon trials for each chronotype group. Effect size analyses demonstrated all chronotype groups performed better in the morning compared to afternoon (*small*-*large* effects). Likewise, there were nonsignificant differences (*p* >0.05) in shooting scores between chronotype groups in the morning and afternoon trials. Effect size analyses showed M-types scored higher (*moderate* effects) than E-types in morning and afternoon trials, while N-types scored higher than E-types and M-types in the morning (vs. E-types: *large* effect; vs. M-types: *small* effect) and afternoon (vs. E-types: *large* effect; vs. M-types: *moderate* effect) (Table 3).

**Table 3:** Median (inter-quartile range) morning and afternoon trial scores during the modified Basketball Jump Shooting Accuracy Test according to chronotype group.

Trial	Chronotype group			p-value, Cohen’s R		
	M-type (n = 4)	N-type (n = 4)	E-type (n = 5)	M vs N	M vs E	N vs E
***Morning***	213.0 (204.3-223.0)	218.0 (212.3-223.5)	206.0 (196.0-209.0)	0.89, -0.10	0.41, 0.33	0.63, 0.62
***Afternoon***	201.5 (198.5-208.0)	211.5 (208.0-214.8)	191.0 (190.0-202.0)	0.34, -0.36	0.29, 0.41	0.11, 0.53
**p-value, Cohen’s R**	0.47, 0.70	0.47, 0.90	0.72, 0.19			

*Note*: Wilcoxon signed-rank tests were used to assess differences between timepoints for each chronotype group; Kruskal-Wallis tests (with Mann-Whitney U tests as post hoc tests) were used to assess differences between chronotype groups at each timepoint. *Abbreviations*: M = morning-type players; N = neither-type players; E = evening-type players.

## DISCUSSION

The main aims of this pilot study were to 1) explore diurnal variations in shooting accuracy according to player chronotype and 2) compare shooting accuracy between chronotype groups at different times of the day in professional basketball players. The key findings were shooting accuracy was consistent across morning and afternoon trials irrespective of player chronotype, and player chronotype exerted nonsignificant effects on shooting accuracy in the morning and afternoon.

This is the first study to examine the effect of player chronotype on sports-specific skills in professional basketball players. There were non-significant differences in shooting accuracy between morning and afternoon trials across chronotype groups despite effect size analyses showing favourable performance in the morning for each chronotype (M-types: R = 0.70; N-types: R = 0.90; E-types: R = 0.19). Furthermore, non-significant differences in shooting accuracy were evident between chronotype groups at each timepoint, with similar effect size magnitudes evident across morning and afternoon trials for each pairwise comparison (M-type vs. N-type: R = 0.10 to 0.36; M-type vs. E-type: R = 0.33 to 0.41; N-type vs. E-type: R = 0.53 to 0.62) These results contrast the hypothesis that diurnal variations in shooting scores would emerge according to chronotype (i.e. higher scores would be achieved at the timepoint suited to the chronotype group being tested) and in comparisons between chronotypes (i.e. effect magnitudes would fluctuate in favour of chronotypes suited to the time-of-day being tested). Furthermore, our findings contrast previous data in other sports indicating diurnal variations exist in skill performance predominantly examining N-type athletes with tennis first serve accuracy ([16:30-18:00h];^15,16^), badminton serving accuracy ([14:00h]; ^17^), and soccer chipping, volleying ([16:00h, 19:00-21:00h];^9,10^), and dribbling execution^9^ being significantly (*p* <0.05) better in the afternoon and evening compared to the morning (07:00-09:00h). The contrast between the current findings and those provided in previous research may be attributed to the participants recruited in each study. In this regard, the current study examined professional basketball players compared to previous studies that examined amateur^9,10,15,16,17^ and semi-professional athletes^16^. Indeed, professional athletes likely possess a higher degree skill mastery specific to their chosen sports compared to lower-level athletes, which may make them more resistant to diurnal variations in skill execution. However, the notion that professional athletes may be more resistant to diurnal variation compared to lower-level athletes can only be speculated as research on this topic examining professional team sport athletes is only in its infancy.

It may also be plausible that diurnal variations and subsequent daily peaks in skill performance may vary between sports and between skills within each sport^9,10,16^. For example, compared to sports involving greater reliance on gross motor skills, execution of skills in sports involving greater reliance on fine motor control and cognitive demand may be optimal earlier in the day given they are more heavily impacted by the time an athlete has been awake and their subsequent alertness^10^.

While non-significant differences in shooting scores were evident between timepoints for each chronotype group, the *smalllarge* effects favouring morning performance in each chronotype group alludes to the possible effects of the playing sample’s habitual training times. Indeed, the players examined typically completed semi-daily training sessions (up to 4 sessions per week) in the morning, consistently exposing them to early wake times. In contrast, only games were consistently completed in the afternoon or evening (1-2 games per week), meaning performances at later times in the day were habitually completed less often across the season. Exposure of players to early wake times in completing morning training sessions may phase advance the circadian rhythms of N-types and E-types to that indicative of M-types^30^. It has been established that chronotype can be modulated to phase advance or delay acrophases based on the effect of exogenous (e.g. light-dark cycle, temperature; ^30^) and endogenous (e.g. circadian rhythm, adjustment to time zone) factors^14^. Accordingly, it would be reasonable to presume if the same training time for all players resulted in a phase advanced circadian rhythm of N-types and E-types to emulate that of M-types, a null effect of chronotype on shooting accuracy would result. In this way, the time of day at which an athlete habitually trains has only recently begun to be investigated as a mechanism to shift the acrophase of peak performance to that when training takes place with Rae, Stephenson^2^ reporting ~70% of swimmers (n = 26) performed significantly (*p* <0.05) better in the trial that aligned to the time at which they habitually trained (morning vs. afternoon). While this phenomenon was not directly investigated in the current study, the effects observed lend support for the habitual training time of players contributing to the reported findings. However, further research is needed investigating the effect of habitual training times on sport-specific skills in basketball and other team sports to validate this supposition.

It is acknowledged that the key limitation of this study is the sample size distribution across chronotype groups (i.e. <5 players). However, only a single professional basketball team was able to be recruited given the additional travel, costs, and labour, and difficulties associated with recruiting multiple teams from the same league. The single-team recruitment therefore limited the number of definite M-type and E-type players included, with a tertile split approach taken to ensure an even distribution of players across groups in the current study. This approach has been adopted in other chronotype studies examining athletes given the limited M-types and E-types prevalent^31,32^. Furthermore, the small sample size did not allow for players to be matched on key aspects across groups, such as playing position, experience, and shooting ability, which made between- group comparisons difficult to interpret at each timepoint. The small sample size also reduced the statistical power in comparisons made. A second limitation of the current study is that players were only assessed at two time points across the day. Provided the busy training schedules of professional basketball players, the scheduled afternoon trial (15:00-16:30h) was the most practical time to consistently assess players later in the day while the morning trial time (8:00- 9:30hr) overlapped with the typical training time for the recruited players. It would be beneficial to repeat this study by assessing larger samples of professional basketball players across more time points including the typical time that games scheduled at night are played (>19:00h). Finally, it is acknowledged that due to player availability, some players could only complete the afternoon trial on the same day that morning training sessions were completed (>4 hr following training), while other players completed the afternoon trial on days without any scheduled training. While the residual fatigue from training in the morning may have impacted the performance of players in the afternoon trial, there was a similar number of players completing afternoon trials following morning training across groups to nullify this effect (M-types: n = 2; N-types: n = 3; E-types: n = 2).

### Practical Applications

It appears player chronotype does not affect shooting accuracy in professional, male basketball players with shooting performance remaining consistent across morning and afternoon trials in each chronotype group. Alternatively, habitual training time may exert a greater effect on basketball shooting accuracy. From a practical perspective, basketball coaching staff may be permitted to schedule training sessions involving shooting tasks at preferred times across the day with confidence that shooting performance will not exhibit diurnal variations between morning or afternoon sessions. However, coaches may endeavour to match habitual training times with that of games to ensure greatest specificity and align player circadian rhythms to competition.

## CONCLUSION

There were non-significant differences in shooting accuracy between morning and afternoon trials irrespective of player chronotype nor between chronotype groups at each timepoint. However, *small*-*large* effects in shooting accuracy were evident favouring the morning trial across chronotype groups, suggesting habitual training times may exert a prominent effect on shooting accuracy in a team setting. Further research examining wider basketball player samples is encouraged to investigate the interrelating effects of chronotype and habitual training time on skill performance in basketball players.
